# The effect of constitutive pigmentation on the measured emissivity of human skin

**DOI:** 10.1371/journal.pone.0241843

**Published:** 2020-11-25

**Authors:** Matthew Charlton, Sophie A. Stanley, Zoë Whitman, Victoria Wenn, Timothy J. Coats, Mark Sims, Jonathan P. Thompson

**Affiliations:** 1 Department of Cardiovascular Sciences, University of Leicester, Leicester, United Kingdom; 2 Diagnostic Development Unit, University of Leicester, Leicester, United Kingdom; 3 University Hospitals of Leicester NHS Trust, Leicester, United Kingdom; 4 Space Research Centre, School of Physics and Astronomy, University of Leicester, Leicester, United Kingdom; Oak Ridge National Laboratory, UNITED STATES

## Abstract

**Background:**

The measurement of body temperature has become commonplace in the current COVID-19 pandemic. Body temperature can be measured using thermal infrared imaging, a safe, non-contact method that relies on the emissivity of the skin being known to provide accurate readings. Skin pigmentation affects the absorption of visible light and enables us to see variations in skin colour. Pigmentation may also affect the absorption of infrared radiation and thus affect thermal imaging. Human skin has an accepted emissivity of 0.98 but the effect of different skin pigmentation on this value is not known. In this study, we investigated the influence of different skin pigmentation on thermal emissivity in 65 adult volunteers.

**Methods:**

A reference object of known emissivity (electrical tape) was applied to participant’s skin on the inner upper arm. Tape and arm were imaged simultaneously using a thermal infrared camera. The emissivity was set on the camera to the known value for electrical tape. The emissivity was altered manually until the skin temperature using thermal imaging software was equal to the initial tape temperature. This provided the calculated emissivity value of the skin. Participants were grouped according to skin pigmentation, quantified using the Fitzpatrick skin phototyping scale and reflectance spectrophotometry. Differences in emissivity values between skin pigmentation groups were assessed by one-way ANOVA.

**Results:**

The mean calculated emissivity for the 65 participants was 0.972 (range 0.96–0.99). No significant differences in emissivity were observed between participants when grouped by skin pigmentation according to the Fitzpatrick scale (p = 0.859) or reflectance spectrophotometry (p = 0.346).

**Conclusion:**

These data suggest that skin pigmentation does not affect thermal emissivity measurement of skin temperature using thermal infrared imaging. This study will aid further research into the application of thermal infrared imaging as a screening or bedside diagnostic tool in clinical practice.

## Introduction

Temperature measurement is important for medical diagnosis and treatment, and has become much more widely used during the COVID-19 pandemic. Infrared thermography (IRT) provides a non-contact, highly sensitive and accurate measurement of skin surface temperature and its distribution [1–3]. Many potential medical applications of IRT exist, including detection of breast cancer, assessment of diabetic foot complications and mass screening for fever [3]. The pattern of temperature distribution and skin perfusion may also play an important role in the diagnosis and prognostication of critical illness [1].

Any object with a temperature above absolute zero emits electromagnetic radiation, known as infrared or thermal radiation. This emitted radiation is detected by IRT imaging systems to generate temperature readings and a visual representation of the temperature distribution across a surface. To calculate the temperature of an object using IRT, the emissivity of that object must be known. Emissivity describes the efficiency with which an object absorbs and emits radiation at a given temperature when compared to a black body (a ‘perfect emitter’). Real-world objects are not perfect emitters as unless the object is opaque some radiation is reflected and/or transmitted, and therefore have emissivity values of less than one. Transmitted and reflected radiation do not relate to the object’s temperature and must be accounted for to provide an accurate measurement.

Several small studies have attempted to calculate the emissivity of human skin [2, 4–11]. Published emissivity values have varied, probably because of methodological differences between studies. Despite there being no definitive consensus for normal values, most report a range between 0.97 and 0.99, with 0.98 being the most widely accepted figure [2].

Most previous studies have calculated skin emissivity by directly measuring emitted infrared radiation in combination with either skin temperature measurements and a reference point of known emissivity. However, some studies calculated skin emissivity by first determining its reflectivity [5, 10–12].

Despite understanding of the relationship between reflectivity and emissivity, there has been little research into the factors affecting skin reflectivity, and by default skin emissivity. Bernard *et al*. demonstrated a difference in skin surface temperatures measured using IRT before and after the application of commonly encountered substances in medical practice (water, hydration cream, ultrasound gel) [13]. The measurement differences were attributed to the effect the applied substances had on the reflectivity and therefore emissivity of the skin [13].

It has been suggested that skin pigmentation may cause variations in emissivity because of differences in reflectivity [2]. Darker skin tones, if a reduced reflectivity was presumed, may potentially appear cooler than paler skin tones at the same temperature when measured with IRT. This has implications for the application of IRT in clinical practice.

The aim of this study was to quantify the relationship between skin pigmentation assessed with reflectance spectrophotometry, emissivity and measured skin surface temperatures using IRT.

## Materials and methods

### Ethics

After approval by the Research Ethics Committee and Health Research Authority (IRAS 244317/REC 19/HRA/0240), healthy adult volunteers were approached to participate in the study and written informed consent obtained.

### Study design

This was a prospective observational study, carried out at the Diagnostic Development Unit (DDU), based at the Leicester Royal Infirmary, Leicester, UK. All study activity took place in an air-conditioned laboratory based in the DDU. Air temperature within the room ranged from 28–30°C and humidity between 19–30%.

All participants were recruited from healthy staff members in the Emergency and Critical Care Departments at the Leicester Royal Infirmary.

### Data acquisition

**Fig 1:**
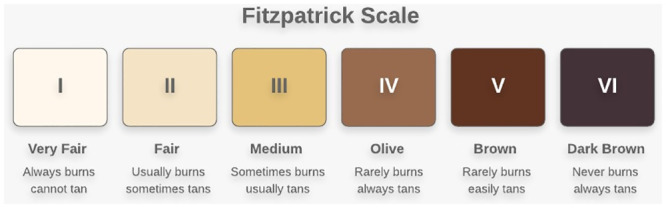
The Fitzpatrick skin phototype scale.

Volunteers’ characteristics including age, weight and ethnicity were recorded. Ethnicity was categorised according to the 2011 population census of England and Wales [14]. Participants were asked to complete a simple questionnaire on genetic factors and skin tanning habits to determine their subjective Fitzpatrick skin phototype (FST) group. The Fitzpatrick classification system (Fig 1) is the method used most commonly to measure constitutive skin pigmentation (i.e. that which is genetically determined and unaffected by ultraviolet exposure). The scale comprises six groups.

**Fig 2:**
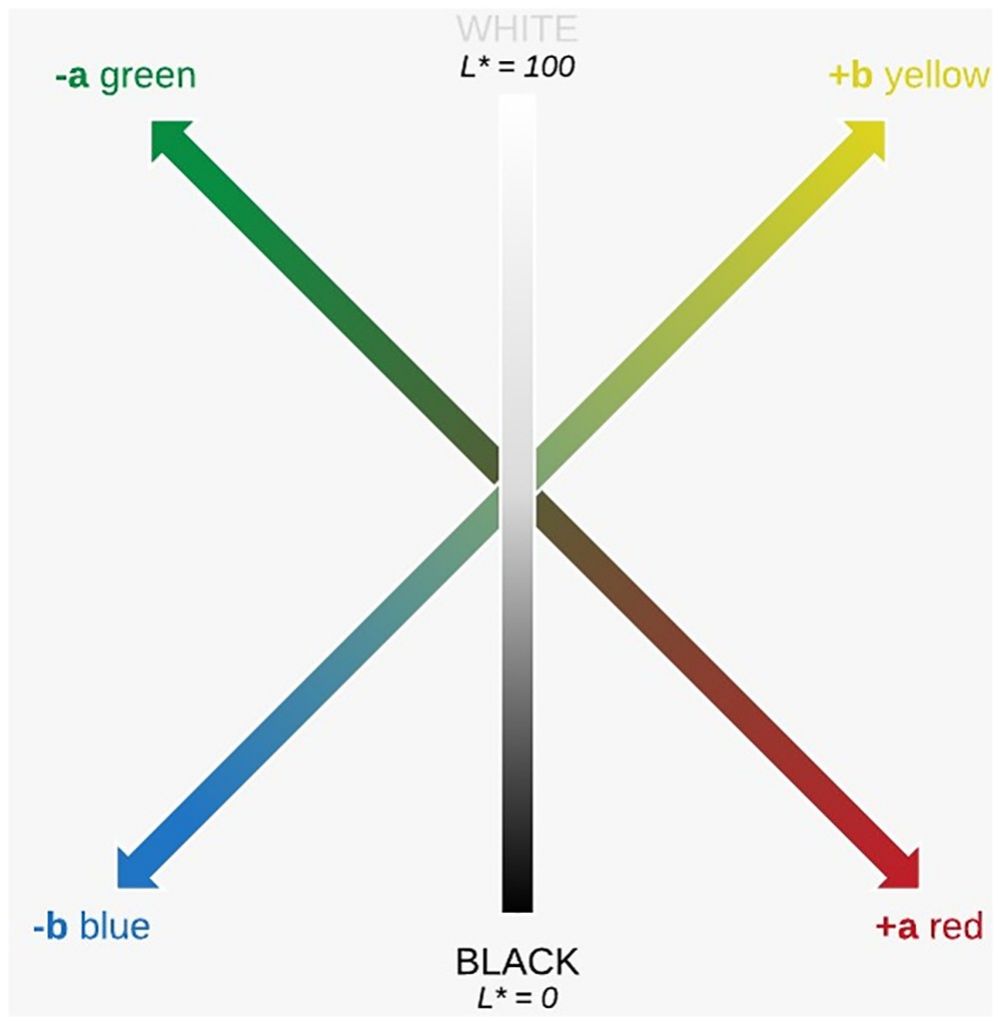
The CIELAB colour space.

Skin pigmentation can be quantitatively measured using reflectance spectrophotometry (RS). In RS, white light is emitted from a standardised source (xenon lamp) onto the target object. Reflected light from the object is recorded at fixed wavelengths in the visible spectrum via a monochromator (400–700 nm), usually at 5–10 nm intervals. Reflected light is expressed as a colour in the L*a*b* (CIELAB) colour space (Fig 2), an international standardised method of colour representation.

**Fig 3:**
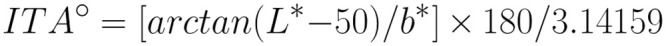
Equation used to calculate the individual typology angle using L* and b* values from the CIELAB colour space.

The CIELAB colour space is displayed on three axes. The L* axis represents lightness (or luminosity), with higher L* values representing lighter shades (L* 100 = white, L* 0 = black). The a* axis represents a continuum between red (+a*) and green (-a*), and the b* axis between yellow (+b*) and blue (-b*). Integrating the L* and b* colorimetric parameters determines the individual typology angle (ITA°) (Fig 3). The ITA° has been shown to correlate well with constitutive skin pigmentation and enable the objective grouping of skin types (Table 1) [15].

**Table 1:** Skin colour categories as proposed by Del Bino et al. based on individual typology angle (ITA°) calculated from reflectance spectrophotometry measurements.

Skin Colour Category	Individual Typology Angle (ITA°)
Very Light	ITA° > 55
Light	41 < ITA° ≤ 55
Intermediate	28 < ITA° ≤ 41
Tan	10 < ITA° ≤ 28
Brown	-30 < ITA° ≤ 10
Dark	ITA° < -30

Skin colour was measured quantitatively using a handheld CM-700d spectrophotometer (Konica Minolta) with an 8mm aperture. Three measurements were taken from the inner surface of the upper-arm and automatically averaged to provide L*a*b* values.

A contact skin temperature probe (M1024222 Skin Temperature Probe, CareFusion, Finland) was applied to the inner surface of the upper arm, along with a 2cm piece of 3M Scotch 88 electrical tape. This tape has a known emissivity value of 0.96 in both the short and long infrared wavelengths [16]. Both the skin temperature probe and tape were covered with a simple ‘stockinette’ tubular bandage and allowed a minimum of 30 minutes to warm to body temperature, with unpublished pilot data demonstrating 5 minutes to be sufficient time to allow the electrical tape and skin temperature to reach thermal equilibrium.

**Fig 4:**
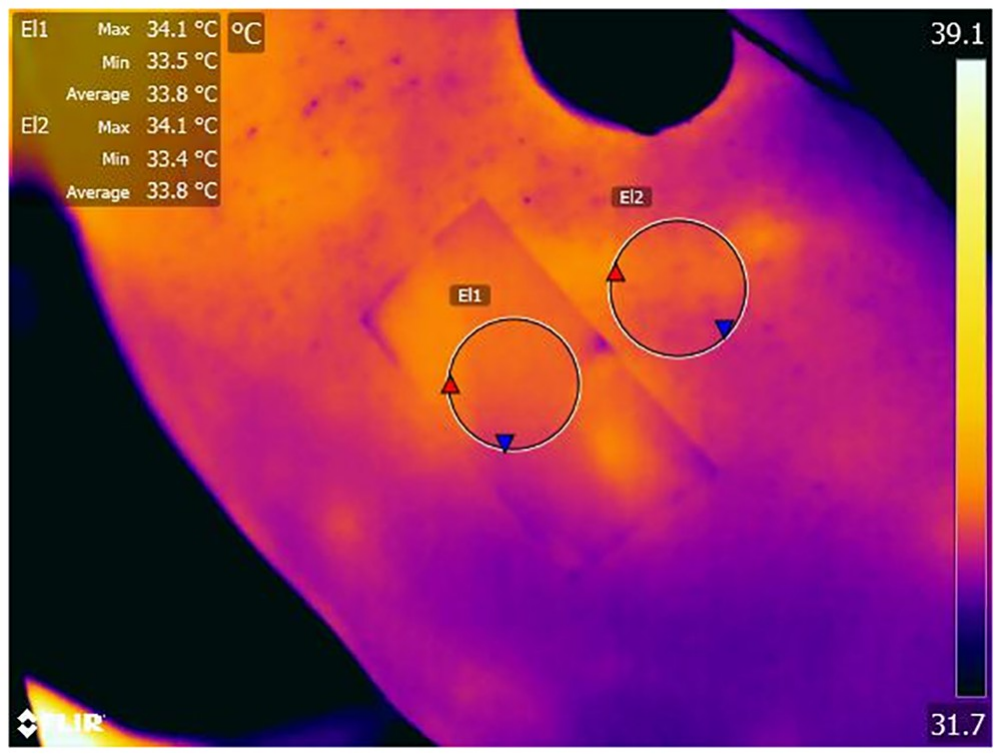
A thermal image of a participants arm with electrical tape in situ (El1).

The stockinette was removed and skin temperature measured. An IRT image of the inner surface of the upper arm was captured under standardised conditions in a single location using a FLIR T650 sc (FLIR Systems Inc.) thermal imaging camera (Fig 4).

### Data analysis

Participant characteristics, L*a*b* values, temperature measurement and FST questionnaire results were initially entered on to individual paper case report forms. Data were then transferred to Microsoft Excel (Office 365, Microsoft) and SPSS Statistics (v24, IBM) for analysis. FST grouping is presented in Fig 1. Individual typology angle (ITA°) was calculated using the formula described by Del Bino et al., and participants grouped accordingly (Table 1).

Thermal images were analysed using FLIR Tools+ Software (v5.13, FLIR Systems Inc.). With emissivity set at 0.96, an average temperature of the tape was measured (T1). This was compared to the average temperature of the adjacent skin (T2). Average temperature values were used in calculation to account for temperature inhomogeneity within the regions of interest (skin and electrical tape). Emissivity was then manually altered until T2 was equal to T1. This was the calculated emissivity of the skin.

### Statistical analysis

Basic descriptive statistics including mean, median and standard deviations were calculated. Inter-group differences were assessed by one-way ANOVA for the FST and ITA° groups. As no data were available to perform a power calculation, a convenience sample of at least 60 participants was chosen.

## Results

In total, 67 participants were recruited to the study. One participant withdrew following informed consent. The thermal images from one participant were lost because of equipment failure. The remaining 65 participants were included in the analysis.

Participants were mostly female (70.8%, n = 46); median age 29 years (range 19–68 years) and median weight 70.5 kgs (range 36–121 kgs). Ethnicity data are shown in Table 2.

**Table 2:** Self-reported ethnicity of participants into categories outlined in the 2011 population census of England and Wales.

Ethnicity, n (%)		
White	British	43 (66.2%)
	Other (Australian/American/New Zealand)	4 (6.2%)
Mixed	White & Black Caribbean	3 (4.6%)
	White & Asian	1 (1.5%)
Black	African	6 (9.2%)
	Caribbean	1 (1.5%)
Asian	Filipino	1 (1.5%)
	Indian	5 (7.7%)
	Pakistani	1 (1.5%)

The majority (69.2%) of participants identified themselves as Group 3 or 4 on the self-reported Fitzpatrick Skin Phototyping (FST) scale. Individual typology angle (ITA°) measurement placed most participants (55.4%) in the *‘very light’* and *‘light’* groups (Tables 3 and 4).

**Table 3:** The distribution of participants according to Fitzpatrick Skin Phototyping (FST) group and the mean calculated emissivity for each FST group.

FST Group	n (%)	Mean Emissivity ± SD (95% confidence interval)
1	3 (4.6%)	0.970±0.010 (0.945–0.995)
2	9 (13.8%)	0.973±0.011 (0.965–0.982)
3	22 (33.8%)	0.973±0.010 (0.968–0.977)
4	23 (35.4%)	0.972±0.010 (0.968–0.976)
5	7 (10.8%)	0.969±0.007 (0.962–0.975)
6	1 (1.5%)	0.980

**Table 4:** The distribution of participants according to individual typology angle (ITA°) group and the mean calculated emissivity for each ITA° group.

ITA° Group	n (%)	Mean Emissivity ± SD (95% confidence interval)
Very Light	19 (29.2%)	0.970±0.009 (0.968–0.974)
Light	17 (26.2%)	0.974±0.011 (0.968–0.979)
Intermediate	10 (15.4%)	0.977±0.013 (0.968–0.986)
Tan	10 (15.4%)	0.971±0.007 (0.966–0.976)
Brown	8 (12.3%)	0.969±0.006 (0.963–0.974)
Dark	1 (1.5%)	0.980

The mean calculated emissivity for all study participants was 0.972 (range 0.96–0.99). The descriptive emissivity statistics for the FST and ITA° groups are given in Tables 3 and 4 respectively.

There were no statistically significant difference between the groups (one-way ANOVA) for either the FST groupings (*F*(5,59) = 0.382, *p* = 0.859), or the ITA° groupings (*F*(5,59) = 1.147, *p* = 0.346).

## Discussion

In this study we found that the mean calculated emissivity did not vary significantly between individuals with different degrees of skin pigmentation.

There are limited data on the relationship between skin tone and emissivity. A few small studies, with sometimes only a single white comparator, have investigated absolute skin emissivity values in participants or skin samples with different pigmentations [4, 6, 11]. Coincidentally these studies observed skin emissivity to be unaltered by skin pigmentation.

It was hypothesised that pigmentation would affect skin reflectivity and therefore emissivity in the infrared spectrum. However, this study contradicts that suggestion. The original hypothesis was based on the effect of pigmentation on light in the visible spectrum that enables us to see variations in skin colour [5, 17, 18]. The layer of the skin responsible for pigmentation is the *stratum basale*, which lies beneath the translucent *stratum corneum* [18]. Although electromagnetic radiation at shorter wavelengths (e.g. visible light - λ = 380nm-780nm) can penetrate the stratum corneum, radiation at longer wavelengths (e.g. infrared - λ = 0.76μm-1000μm) may not. Emissivity to infrared light may therefore be determined by the *stratum corneum*, which is universally translucent among skin of all pigmentations [5, 18]. The effect of wavelength on reflectivity in skin of different pigmentation has been investigated experimentally. Two small studies demonstrated that the proportion of radiation absorbed by darker skin tones decreases with increasing wavelength to a point where it is equivalent regardless of pigmentation [10, 19]. Jacquez et al. estimate this to be at approximately λ = 1.2μm [10]. Overall this suggests that in the infrared spectrum skin reflectivity, and therefore emissivity, is unaffected by pigmentation and concurs with the findings of the current study.

The mean calculated emissivity for the skin of all participants in this study was 0.972.

This is consistent with existing literature that accepts a value of between 0.97 and 0.99 [2].

This is the largest study in this area and has used an improved method to measure emissivity, objectively quantifying the level of skin pigmentation and a larger sample size. We used an existing method involving comparison to a reference object of known emissivity [4, 6–9]. In previous studies, the temperature of bulky reference and target objects had to be separately measured and controlled. Small measurement variations had the potential to substantially affect emissivity calculations as the value is so close to unity [4, 6–9]. We addressed this problem by using a reference object (electrical tape) applied directly to the target object (skin) with continuous real-time temperature measurement of both objects using the same temperature probe.

This study aimed to investigate any potential variation in calculated emissivity caused by differences in skin pigmentation. The methodological aim was therefore to preserve true variation and minimise confounding sources of variation. Hair density is one possible confounder. Hair is avascular meaning it often appears cooler on IRT images [2, 20]. It is possible that unknown variations in hair density between skin tone groups may explain these results although this is unlikely as the area of the body used in this study (inner upper arm) is typically of low hair density. Substances applied to the skin have also been shown to affect emissivity and therefore apparent temperature [13]. Again, it is unlikely that makeup or other substances would have been applied to the inside of the upper arm. Considering that the long-term application of this work relates to IRT use in clinical practice or non-clinical situations, using samples that do not control for these potential confounders may be more representative of the eventual target population.

A convenience sample was used in this study, as no existing data were available to perform a power calculation. Although the study is larger than previous studies, it is potentially underpowered. The FST scale, although useful, has several limitations. Constitutive skin pigmentation may be more accurately represented by assessing skin colour in unexposed areas as opposed to the skin’s response to sun exposure. Individuals may also be grouped based on their ethnicity as opposed to their absolute skin pigmentation, with many black individuals being allocated Group 6 when this is not necessarily accurate [20]. Previous studies often involved only one non-white comparator [6]. Our study improves on this by quantifying skin pigmentation and showing that the majority (69.2%) of participants identified themselves as Group 3 or 4 on the FST scale.

Unfortunately, only one volunteer was classified as Group 6 on the FST scale or *‘dark’* on the ITA° classification. The calculated skin emissivity value for this individual was very similar to the other groups, which all contained similar numbers of participants, with no significant variation in emissivity between groups. Recruitment was restricted by the ethnicity and characteristics of our local population, and we could not overcome this limitation of the study. Further studies in areas with different population demographics would strengthen the application of IRT more widely.

## Conclusion

These data show that skin emissivity in humans is unaffected by skin pigmentation and support the use of an emissivity value of 0.98 for universal use. Therefore the emissivity value used to calculate temperature using IRT does not need to be altered based on an individual’s skin tone. This will inform and enable further enable research into IRT in clinical practice and other applications.
